# Comparative Analysis of the Thermal Insulation of Multi-Layer Thermal Inserts in a Protective Jacket

**DOI:** 10.3390/ma13122672

**Published:** 2020-06-12

**Authors:** Dubravko Rogale, Goran Majstorović, Snježana Firšt Rogale

**Affiliations:** 1 Faculty of Textile Technology, University of Zagreb, 10000 Zagreb, Croatia; dubravko.rogale@ttf.hr; 2 Weltex, 11000 Beograd, Serbia; goranmajstor77@gmail.com

**Keywords:** textile materials, thermal insulation, clothing system, multi-layer thermal inserts, thermal manikin

## Abstract

This paper presents the measurement results of the thermal insulation of the outer shell, thermal inserts, and clothing systems, as well as a comparative analysis of the thermal insulation of multi-layer thermal inserts in a thermal jacket intended for professional services in cold weather. Detachable thermal inserts are made of double-faced, diamond-shaped quilted lining with different masses per unit area, and together with the jacket, they form clothing systems with different thermal properties. Tests of the thermal properties of clothing were performed on a thermal manikin. They showed that an increase in the mass of thermal insulation textile materials contributes to an increase in the thermal insulation properties of clothing and are insufficient for a complete analysis of the thermal properties of clothing. Therefore, for the first time, three new parameters of integration efficiency of the thermal insert, thermal insulation efficiency parameters, and efficiency parameters of the integration of the textile material integrated into the clothing system were introduced. Based on these parameters, it is possible to perform an effective and accurate comparative analysis of the thermal insulation of multi-layer thermal inserts in clothing. This makes it possible to apply exact scientific methods largely in the technical design of the thermal properties of integrated textile materials, instead of experience-based methods as in the past.

## 1. Introduction

Exact measurements in the field of thermal insulation of clothing began in the 1940s when the US Army was developing the first thermal manikin [1], and more intensely in the 1980s when international research on cold protective clothing, clothing physiology, and thermal functions of clothing began. Thermal insulation is nowadays expressed in SI-units by m² K W^−1^. Gagge et al. published a scientific paper in 1941 defining a warm business suit providing thermal insulation of approximately 0.155 m² K W^−1^ for the whole body, which was originally equal to 1 Clo unit [2], and refers to a person who feels thermal comfort when sitting in a ventilated room with an ambient temperature of 21 °C, an airflow of 0.1 m s^−1^, and a relative humidity of less than 50%. The Clo, was defined in J. R. Mather’s “Climatology: Fundamentals and Applications” as units measuring the thermal insulation value of clothing, as well. To achieve a more simple perception of these units, it should be pointed out that the naked human body has an insulation value of 0.0 Clo, and a value of 1.0 Clo refers to a person who is dressed in a typical business suit [3,4]. The Clo unit is easier to understand and more used in clothing engineering.

In his work, Eryuruk examined the thermal properties of fabrics and their combinations in a three-layer garment composite. Sixteen garment composites were examined, combining two types of fabrics for making outer shells, four types of moisture barrier fabrics with membranes, and two types of thermal barrier fabrics. It was found that thermal and moisture comfort properties were significantly affected by different fabric layers [5].

Matusiak tested the thermal insulation properties of single-layer and multi-layer textile materials on the Alambeta instrument. Different types of materials intended for the manufacture of winter clothing: Cotton woven fabrics, thermal textile materials, and sets of both materials were examined [6].

Matusiak and Sybilska conducted a study in which they analysed the relationship between the thermal insulation of nine fabrics of different raw material compositions on the Alambeta instrument and the thermal insulation of T-shirts made from the tested fabrics of the same design and size [7]. The authors concluded that it is possible to design garments based on the measurement of the thermal insulation of the material with regard to the necessary thermal protection.

The Alambeta instrument was also used by Gupta et al. in testing the thermal properties of single and double layer fabric assemblies. The effect of layering fabrics with and without air gaps between them has been assessed to simulate the effect of a multi-layered garment assembly. Results show that thermal insulation increases markedly when an inner layer is paired with an outer layer of fabric [8].

Konarska et al. [3] compared the measurement results of the three ensembles of disposable medical clothing obtained by measuring the thermal manikin and subjective assessment of the wearer. On the thermal manikin the values of the heat balance of the thermal insulation of the thermal insert were measured, as well as the thermal insulation of the thermal insert, temperature, and heat losses, while a subjective assessment was used to determine values of dry heat losses under the conditions of heat comfort. The results showed that the thermal insulation value of the tested clothing samples was 13% higher in the subjective assessment of the wearer compared to the thermal manikin tests. It has also been shown that measurements on the thermal manikin are more accurate with regard to the measurements of subjective assessment of the wearer (manikin’s error 2%, subjects’ error 12–18%).

Koranska et al. also investigated the impact as thermal environment parameters, how heating power is transferred to the manikin, and thus required to reach thermal balance during tests with thermal clothing insulation. Tests are carried out on three double sets of cold protective clothing intended for use in very low temperatures. Based on the results, they conclude that methods for controlling the transfer of heating power do not have a major impact on the thermal insulation of clothing, that air velocity decreases thermal insulation, and that the temperature in the air-conditioning chamber should be determined in accordance with the anticipated clothing insulation of the clothing ensemble being tested. The authors of the paper propose changes of the measurement conditions on the thermal manikin prescribed by ISO 15831 [9] in terms of decreasing the admissible range of air velocity to values from 0.3 to 0.7 m s^−1^, as well as increasing the temperature range between the thermal manikin surface and the environmental temperature in proportion to the thermal insulation of clothing [10].

Moreover, Holand dealt with the comparison of the results obtained by measuring on a thermal manikin and subjective assessment of the wearer [11]. He conducted tests of seven different types of sleeping bags, (one military and six commercial sleeping bags). Sleeping bags were tested on a thermal manikin in a climatic chamber where the temperature was adjusted to achieve a total heat loss from the manikin between 40 and 80 W/m^2^ (normally around 55 W/m^2^) when the system thermally ended up in a steady state with the environment. Subsequently, subjective assessment measurements were carried out on test persons who stayed in the climatic chamber (between 11:00 pm and 6:00 am), whereby the ambient temperatures were compared to the expected lower limit of sleeping comfort for different sleeping bags. The author states that the results are fairly equal, and the lower thermal insulation values of individual sleeping bags obtained by the subjective assessment of subjects that justify such cold spots (e.g., cold feet) may be a significant reason for thermal discomfort and consequently poor sleep. In addition, the thermal insulation of certain garments in the men’s clothing system (undershirts, boxer shorts, socks, shirts, jeans, jackets, so-called “peaked caps”, and shoes) and the entire clothing system was examined. This research was carried out on a thermal manikin on different menswear systems according to the international standard ISO 15831. The authors concluded that dressing in layers increases the overall thermal insulation of the clothing system. Exact measurements on the manikin have shown that the use of different clothing combinations can influence the thermal insulation of the clothing system [12].

Oliveira et al. compared the thermal properties of a summer ensemble, a typical business suit, and a cold protective clothing ensemble. Using different methods on a thermal manikin with 16 body parts, they measured the thermal insulation of the thermal insert and concluded that differences between calculation methods can be significant and that greater discrepancies arise when the distribution of clothing becomes less uniform. Other authors reached similar conclusions [13,14].

It is possible to implement new modern structures in the clothes, such as Smart Textile Materials with Shape Memory Alloys, whose task will be to preserve body heat or thermal protection insurance that were not previously used in conventional technical garment construction [15,16].

This brief overview of the use and benefits of measurement systems for testing the thermal properties of clothing, in particular of thermal manikins, shows that they can be very useful in the technical design of the thermal properties of clothing. However, they are still used on a small scale and are largely restricted for scientific research. When designing garments in the garment industry, visual design predominates from the point of view of aesthetics, and technical design, especially from the point of view of the thermal properties of the integrated textile materials, is not applied, but rather experience-based methods are used. Therefore, measurements of the resistance to heat transfer from the human body through the clothing to the environment should be introduced in the process of engineering clothing design. In this way, it is possible to accurately determine the success of designing the thermal properties of clothing and the usefulness of textile materials intended for the thermal insulation of clothing.

The paper deals with the design and practical performance of a professional protective jacket for cold conditions, where the outer shell consists of the base fabric and lining, and there is the possibility of inserting a detachable thermal insert into the inside of the jacket. The measurement results of the thermal insulation of the outer shell, thermal inserts, and clothing systems as a combination of outer shell and different multi-layer thermal inserts are presented.

Based on the measurement results of the thermal manikin, a new concept for the integration of the thermal insert has been presented in this work. Furthermore, a new method and procedure for evaluating thermal insulation efficiency is presented, which indicates how much mass of textile material had to be incorporated into a garment to achieve a thermal insulation effect of 1 Clo (thermal insulation of 0.155 m^2^ K W^−1^). The method and procedure for evaluating the efficiency of the incorporated mass of the textile insulation materials incorporated into the clothing system of the professional protective jacket and the efficiency of the mass of the textile insulation materials integrated into the thermal inserts are also presented. The efficiency of the integrated mass of textile insulation material indicates the value of the achieved thermal insulation when using 1 kg of textile insulation material. The measurements, the obtained results, and the adopted method presented in this paper confirm the thesis that it is necessary to abandon the current practice of the empirically determined selection of types and mas per unit area of textile materials intended for thermal insulation of clothing, and that modern measurement technology and scientific methods should be used in the engineering design of thermal properties of clothing. In assessing the characteristics of thermal parameters of protective clothing and uniforms for special services, when, following an invitation for tenders, garments are delivered by several different manufacturers using different garment cuts, materials, raw material compositions, and combinations of built-in composites (base fabrics, reinforcements, adhesive interlinings, and linings), the exact possibility of assessing the garment from the point of view of the precisely measured degree of thermal protection is not used. The paper presents a new method of technical design and construction of protective clothing from the point of view of thermal insulation, which enables a more reliable, faster, and technically accurate design and construction. This confirms the hypothesis that not only previous experience-based methods of clothing design can be used, but also more complex methods of technical clothing design. The tests of the thermal properties of clothing were carried out with a thermal manikin using the series method in a static mode according to ISO 15381.

## 2. Materials and Methods

**Figure 1:**
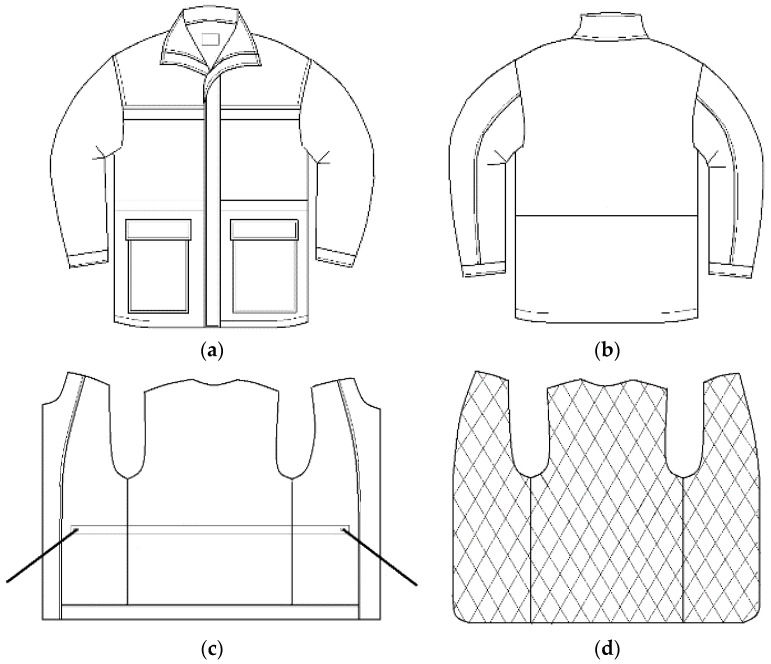
Basic construction features of the test garment. (**a**) The front of the outer shell; (**b**) the back of the outer shell; (**c**) lining; (**d**) detachable thermal insert.

**Figure 2:**
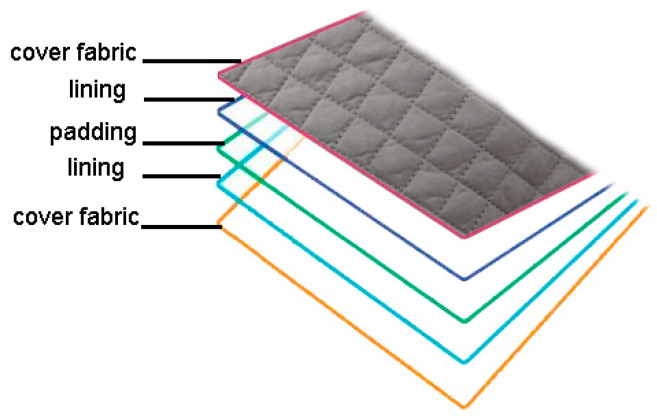
Double-faced, diamond-shaped quilted lining.

The professional protective jacket (clothing systems) consists of an outer shell made of outer water repellent fabric (designation M01), lining fabric (designation M02), and five types of different thermal inserts (designations TI1 to TI5) as detachable thermal inserts with different masses of textile thermal insulation materials (Figure 1). Thermal inserts are made in five combinations of double-faced, diamond-shaped quilted lining (M03 to M07) with different masses per unit area. The four combinations consist of five layers: Two layers of cover fabrics, two layers of lining and one layer of padding, and a combination of nine layers: Four layers of cover fabrics, four layers of lining, and one layer of padding (Figure 2). The four five-layer combinations differ in weight from padding amounting to 0.385 g m^−2^ (designation M03), 0.790 g m^−2^ (designation M04), 0.114 g m^−2^ (designation M05), and 0.145 g m^−2^ (designation M06) (Table 1). Five clothing systems (designations CS1 to CS5) with professional jackets made for cold conditions, are shown in Table 2. The fifth combination of thermal inserts consists of two layers of a double-sided, diamond-shaped quilted lining with the designation M03. Table 2 shows the designations of the materials used to make the outer shell, thermal inserts, and clothing systems, and gives the values of their masses.

The thermal insulation properties of a protective jacket for professional services without integrated thermal inserts were tested, then for thermal inserts made of double-sided, diamond-shaped quilted lining with different surface masses per unit area, and finally for five garment systems consisting of an outer shell and thermal inserts. Tests of thermal properties were performed on the thermal manikin.

**Figure 3:**
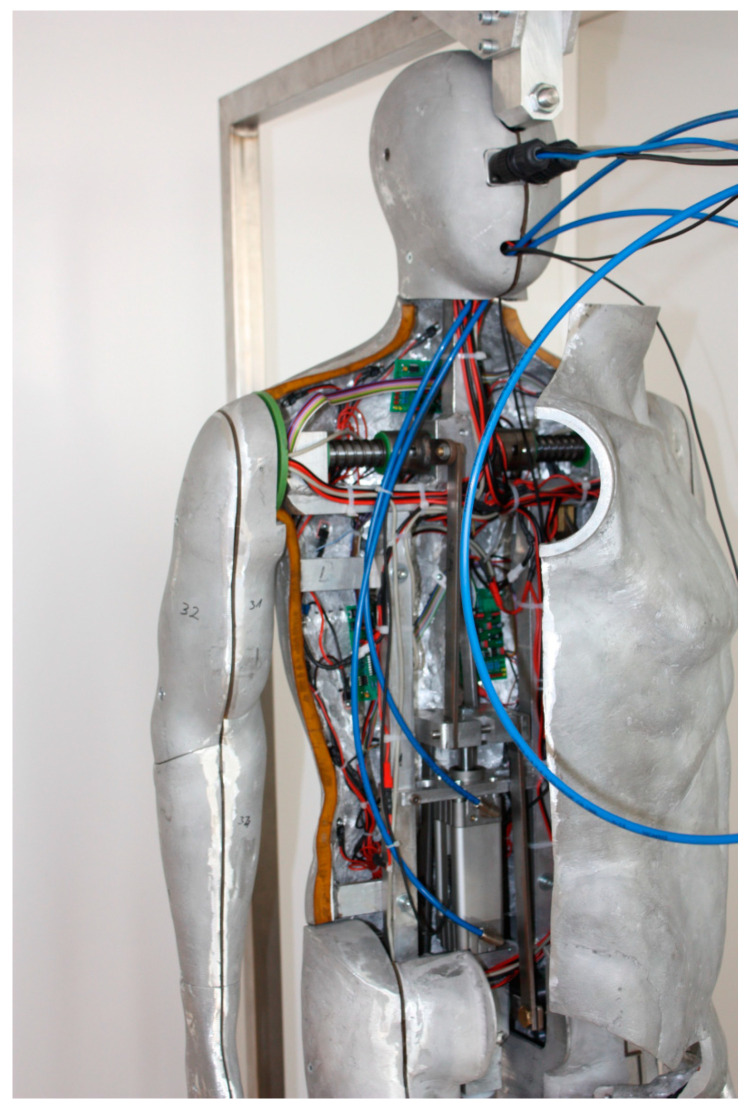
Thermal manikin.

Testing of thermal properties on a thermal manikin was performed using the serial method in static mode according to ISO 15381, Figure 3, implemented, installed, calibrated, and patented at the Faculty of Textile Technology of the University of Zagreb [17].

After establishing the specified environmental conditions in accordance with ISO 15381 for the control and measurement of ambient air parameters (temperature, relative humidity, and flow velocity), the thermal insulation of the empty surface of the manikin together with the air boundary layer along the surface of the manikin R_ct0_ is determined in the measuring system.

(1) $$R_{\mathrm{ct}0}=\frac{\left(T_{s}-T_{a}\right)\cdot A}{H_{0}}$$

where R_ct0_ is the resultant total thermal insulation of the measuring device including the thermal insulation of the boundary air layer (m^2^ kW^−1^); A is the surface area of the body segment i of the manikin (m^2^); Ts is the mean skin surface temperature of the manikin (°C); Ta is the air temperature within the climatic chamber (°C), and H_0_ is the total heating power supplied to the manikin (W).

The composite specimen to be tested is placed on a measuring unit of an electrically heated plate after the resultant total thermal insulation of the measuring device is determined and the heat flux through the test specimen is measured after new stable conditions have been reached. The assessment of thermal properties of clothing by means of the thermal manikin is conducted in such a way that the chosen garment or ensemble is placed around the body of the thermal manikin in the static or dynamic operational mode [18]. After thermal comfort has been established, which can be detected from the stabilization of parameter values, measurements are performed and thermal insulation is calculated from Equation (2):

(2) $$R_{\mathrm{ct}}=\frac{\left(T_{s}-T_{a}\right)\cdot A}{H_{0}}-R_{\mathrm{ct}0}$$

In order to be able to compare the design results of different integration materials, thermal inserts, and clothing systems exactly and accurately, it is necessary to create a mathematical term for calculating the efficiency of thermal insulation. Based on the research conducted so far by a group of authors of this paper, a new term for the efficiency of thermal insulation, EI_CS_, has been formulated, which represents the mass of thermal insulator required for a clothing system to have a thermal insulation of 1 Clo. Thermal insulation efficiency (EI_CS_) is expressed by mathematical expression (3) [19].

(3) $${\mathrm{EI}}_{\mathrm{CS}}=\frac{m_{\mathrm{cs}}}{{\mathrm{Clo}}_{\mathrm{cs}}},\;(\mathrm{kg}\cdot {\mathrm{Clo}}^{-1})$$

where Clo_CS_ is the thermal insulation of the clothing system (Clo); m_CS_ is the mass of the clothing system (kg).

Mathematical expressions were also determined for the calculation of the efficiency of the textile mass installed on the thermal insulation in the technical design of clothing systems. On the basis of the previous research, the original expression for the efficiency of the textile mass integrated into the clothing system was established, which represents the value of thermal insulation achieved with a mass of 1 kg of materials integrated into the clothing system. The efficiency of the textile mass integrated into the clothing system (EM_CS_) is expressed by mathematical expression (4) [19].

(4) $${\mathrm{EM}}_{\mathrm{CS}}=\frac{{\mathrm{Clo}}_{\mathrm{CS}}}{m_{\mathrm{CS}}},\;(\mathrm{Clo}\cdot {\mathrm{kg}}^{-1})$$

## 3. Results and Discussion

Testing the thermal insulation of an undressed thermal manikin in static mode was performed for outer shells and thermal inserts combined in different test clothing systems. Thermal insulation measurements of the test clothing systems on the thermal manikin were performed in the climatic chamber of the measuring system for testing the thermal properties of composites and clothing, in which the thermal insulation of the undressed thermal manikin was also performed. The measurements were performed at an ambient air temperature of 20 °C, a surface temperature of the thermal manikin of 34 °C, and an air flow velocity of 0.4 m s^−1^. Relative humidity ranged from 47–50%. Measurements are made by measuring the temperature of the (heated) segments involved and the power of the heater, and the average values are plotted every minute. The measurement takes 20 min according to ISO 15831.

The measured total thermal insulation of the undressed manikin together with the air boundary layer along the surface (R_ct0_) was 0.09683 m^2^ K W^−1^.

The measured value of thermal insulation of the outer shell (OS1) was 1.08 Clo (0.167 m^2^ K W^−1^).

**Figure 4:**
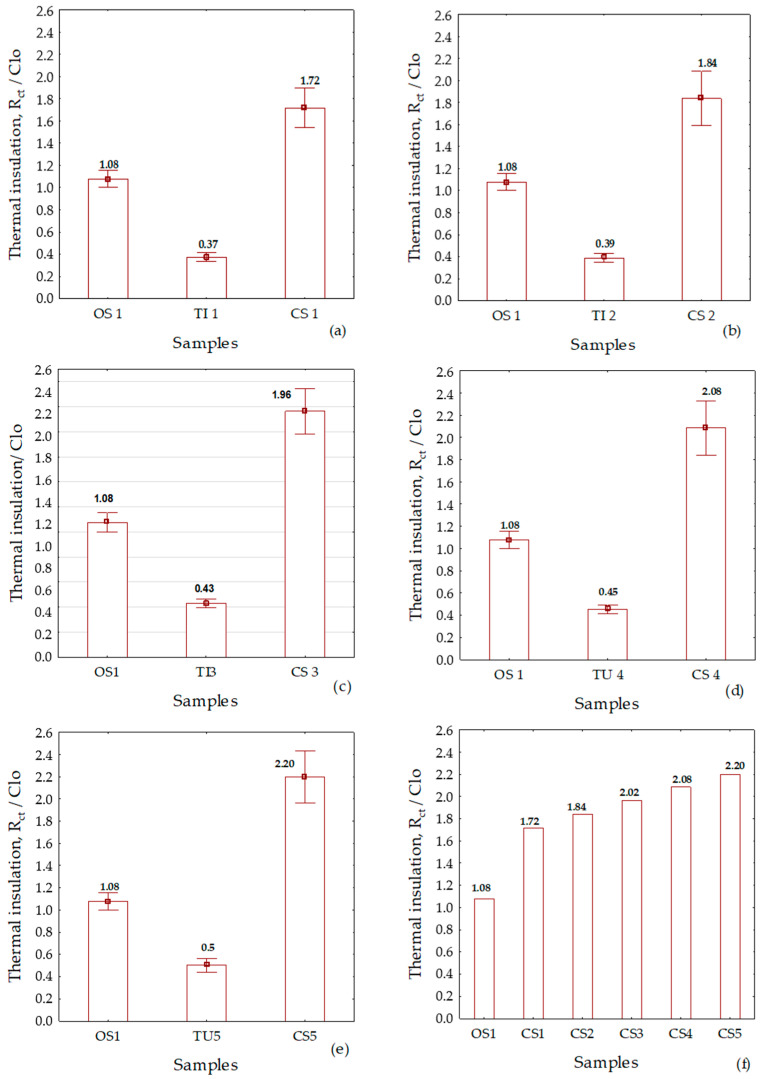
Graphical representations of the measurement results of the thermal insulation of the outer shell, thermal inserts, and clothing systems: (**a**) Clothing system (CS1); (**b**) clothing system (CS2); (**c**) clothing system (CS3); (**d**) clothing system (CS4); (**e**) clothing system (CS5); (**f**) increasing the thermal insulation of the clothing systems by installing thermal inserts of different masses per unit area.

The clothing system (CS1) consists of an outer shell (OS1) and a thermal insert (TI1). The value of the thermal insulation of the thermal insert (TI1) was 0.37 Clo (0.057 m^2^ K W^−1^). After these measurements, the outer shell (OS1) and the thermal insert (TI1) were assembled into a clothing system (CS1) and thermal insulation values of 1.72 Clo (0.266 m^2^ K W^−1^) were measured. Based on the measured data set, a graphical representation of the results was created (Figure 4a).

For the clothing system (CS2), a thermal insert (TI2) was selected in addition to the outer shell (OS1). The value of the thermal insulation of the thermal insert (TI2) was 0.39 Clo (0.060 m^2^ K W^−1^), and the measurement value of thermal insulation of the clothing system (CS2) was 1.84 Clo (0.285 m^2^ K W^−1^). Based on the measured data set, a graphical representation of the results was created (Figure 4b).

For the clothing system (CS3), a thermal insert (TI3) was selected in addition to the outer shell (OS1). The value of the thermal insulation of the thermal insert (TI3) was 0.43 Clo (0.067 m^2^ K W^−1^). After these measurements, the outer shell (OS1) and the thermal insert (TI1) were assembled into a clothing system (CS3) and thermal insulation of the new clothing system (CS3) were measured. The measurement value of thermal insulation of the clothing system (C3) was 2.02 Clo (0.304 m^2^ K W^−1^). Based on the measured data set, a graphical representation of the results was created (Figure 4c).

For the clothing system (CS4), a thermal insert (TI4) was selected in addition to the outer shell (OS1). The value of the thermal insulation of the thermal insert (TI4) was 0.45 Clo (0.070 m^2^ K W^−1^), and the measurement value of thermal insulation of the clothing system (CS4) was 2.08 Clo (0.323 m^2^ K W^−1^). Based on the measured data set, a graphical representation of the results was created (Figure 4d).

For the clothing system (CS5), a thermal insert (TI5) was selected in addition to the outer shell (OS1). The value of the thermal insulation of the thermal insert (TI5) was 0.5 Clo (0.078 m^2^ K W^−1^), and the measurement value of thermal insulation of the clothing system (CS5) (0.341 m^2^ K W^−1^) was measured. Based on the measured data set, a graphical representation of the results was created (Figure 4e).

**Figure 5:**
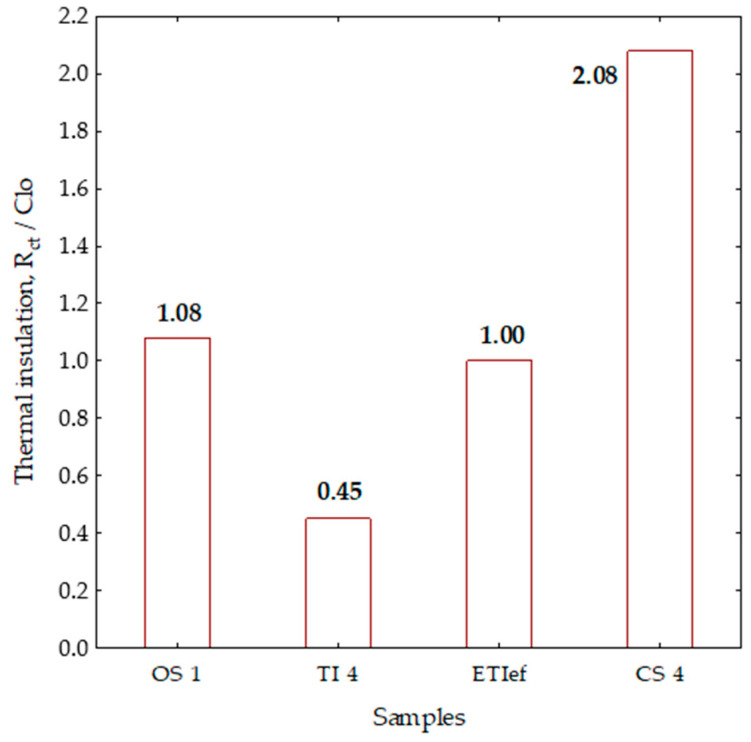
Presentation of the thermal insulation efficiency parameter (ETIef) calculated from the value of the thermal insulations of the clothing system (CS) and the outer shell (OS).

On the basis of the measured values and the analysis of the measurement results on the manikin, the summarized results of the thermal insulation of all clothing systems are shown in Figure 5f. The measurement results for all clothing systems are presented according to the increasing value of the total mass of textile materials intended for thermal insulation. From Figure 4f, the basic conclusion can be drawn that the thermal insulation of the garment increases with the mass of the integrated textile material, which is evidence of an already known empirical fact. This is not enough for a scientific analysis, but it is necessary to approach the definition of new parameters and their comparative analysis to explain the effects of detachable multi-layer thermal inserts in the professional protective jacket for colder conditions.

### 3.1. Defining the Thermal Parameter of the Integration Efficiency of the Thermal Insert

The analysis of the measured data on the thermal manikin for five different garment systems (CS) shown in Figure 4a–e reveals that the value of thermal insulation is greater than the algebraic sum of the measured thermal insulation of the outer shell (OS) and thermal insert (TI). It can be concluded from this that by inserting a thermal insert into the outer shell an additional air layer is formed, which additionally increases the overall thermal insulation properties, which is in any case a positive effect. This effect cannot be measured directly on the manikin, but, as a newly introduced analytical parameter of the integration efficiency of the thermal insert (ETI_ef_), it can be calculated from the values of the thermal insulation of the clothing system (CS) and the outer shell (OS), as shown in Figure 5. The representation is based on the data in Figure 4d for the thermal insert (TI4). A column with the designation (ETI_ef_) for the thermal insulation of the thermal insert and with a thermal insulation value of 1 Clo was determined and plotted as the difference between the total thermal insulation of the clothing system with 2.08 Clo and the thermal insulation of the outer shell (OS1) of 1.08 Clo. The introduction of the integration efficiency parameter (ETI_ef_) defines the effects of the integration of this insert much better because, in addition to the insulation properties of the thermal insert measured on the thermal manikin, it also accurately evaluates the effects of the layer of air created by the integration of the thermal insert in the outer shell. The influence of the newly formed air layer can be determined for the case under consideration from the difference between the integration efficiency (1 Clo) and the value of the measured thermal insulation of the thermal insert on the manikin (0.45 Clo). The identified difference of 0.55 Clo can be attributed to the newly formed air layer between the outer shell and the thermal insert. Based on the defined parameter of integration efficiency of the thermal insert it is possible to calculate and accurately evaluate the thermal impact of the newly created airbag. The way for a comparative analysis of the thermal insulation of multi-layer, detachable thermal inserts is also paved.

**Figure 6:**
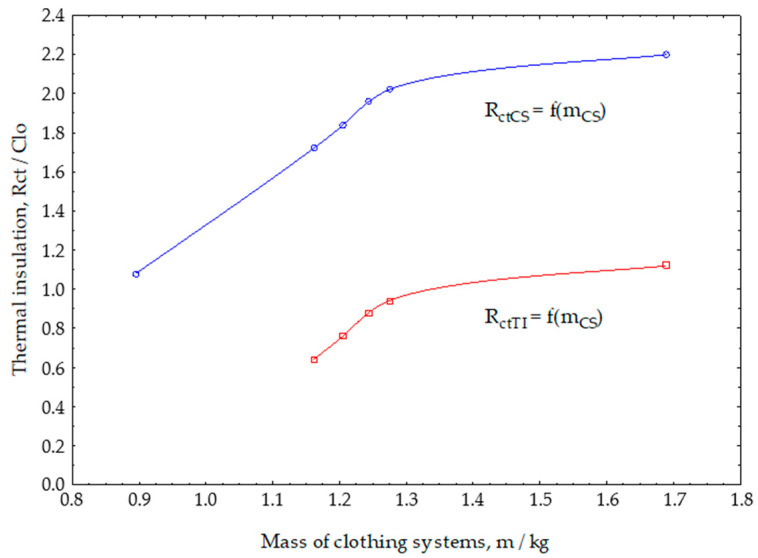
Functional dependence on the value of thermal insulations of clothing systems on the mass of the integrated textile material with thermal insulation properties.

The functional dependence on the thermal insulation value of clothing systems on the mass of the integrated textile material with thermal insulation properties is shown in Figure 6. It is obvious that the thermal insulation of clothing systems increases with the increase in mass of the clothing system, as a functional dependence R_ctCS_ = f (m_CS_). The increase is stronger with smaller masses and then saturation occurs when the increase in the installed mass of thermal inserts does not significantly increase the thermal insulation. This observed effect can be attributed to the occurrence of chimney effect when, due to the increased cross-section of the insulation layer, heat convection caused by a slight air flow through the insulation material reduces the insulation properties. This is one possible reason why people do not wear one thick garment but thinner garments, which is called layered clothing.

Figure 6 also shows the functional dependence on the thermal insulation value of the thermal inserts as a function of the mass of the clothing system. The first functional dependence of R_ctCS_ is the cumulative effect of the outer shell and the clothing system (outer shells with thermal inserts). Functional dependence R_ctTI_ = f (m_CS_) represents the change in thermal properties in the clothing system caused only by the change of mass of thermal inserts.

From both functional dependencies, it can be seen that above a certain mass of thermal inserts, i.e., the mass of the clothing system, there is no significant increase in the value of the thermal insulation. Thus, when technically designing the thermal properties of the garment at a certain limit, it is no longer necessary to increase the mass of the textile thermal insulation material. At this point, the increase in the mass of the garment will not lead to an increase in thermal insulation, and on the other hand, it will cause a heavier garment, increase the price of the garment due to the higher volume of the material that is integrated, while at the same time the bulkiness of the garment hinders the ease of movement.

### 3.2. Results and Discussion of Thermal Insulation Efficiency Parameters and Efficiency Parameter of the Textile Mass Integrated into the Clothing System

In order to determine the limit when a further increase in the mass of the textile thermal insulation material does not contribute to a significant increase in the thermal insulation properties of garments, it is necessary to define new parameters of thermal insulation efficiency and the textile mass integrated into the clothing system. These parameters were also defined for the first time for the purposes of this paper.

**Figure 7:**
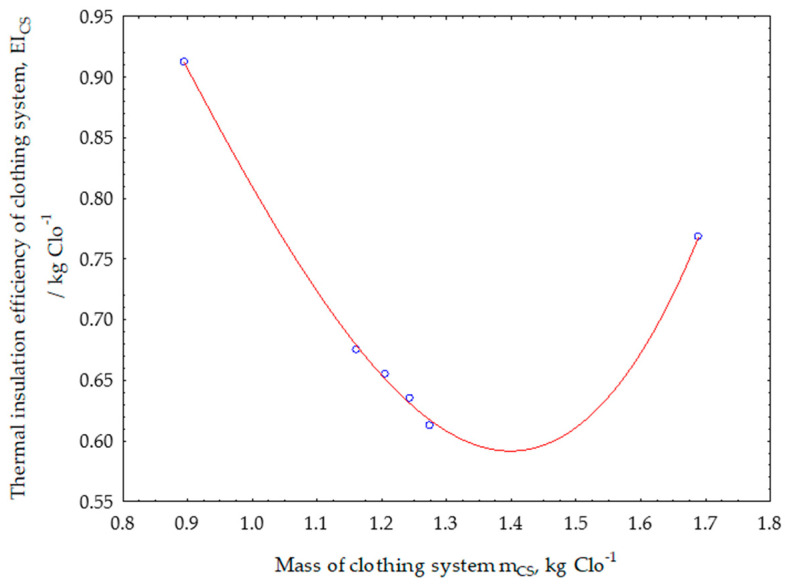
Functional dependence on changes in the values of the parameters of thermal insulation efficiency of clothing systems.

The functional dependence on changes in the values of the parameters of thermal insulation efficiency is shown in Figure 7 and is determined using expression (3).

The thermal insulation efficiency parameter shows how much mass of textile insulation material should be integrated into the garment to achieve a thermal insulation of 1 Clo. Therefore, this parameter is very important in the comparative analysis of the thermal insulation of garments, if technically exact, based on measured values on a manikin, the optimal multi-layer insulation effect can be accurately evaluated. The smaller the amount of textile insulation material required for a certain degree of thermal protection, the more successful the clothing project will be. Figure 7 shows that it is most difficult to achieve adequate thermal protection only by using an outer shell. The addition of detachable thermal inserts into the protective jacket, which is intended for cooler conditions, resulted in a great decrease in the required mass of thermal insulation textile materials.

The curve has a pronounced minimum where the optimum of thermal insulation properties is achieved with a minimum of the integrated mass. Increasing the thermal insulation mass no longer achieves optimum effect, so the minimum of the function shown is the limit beyond which there is no rational reason to increase the mass of textile thermal insulation materials to increase the thermal properties of clothing.

**Figure 8:**
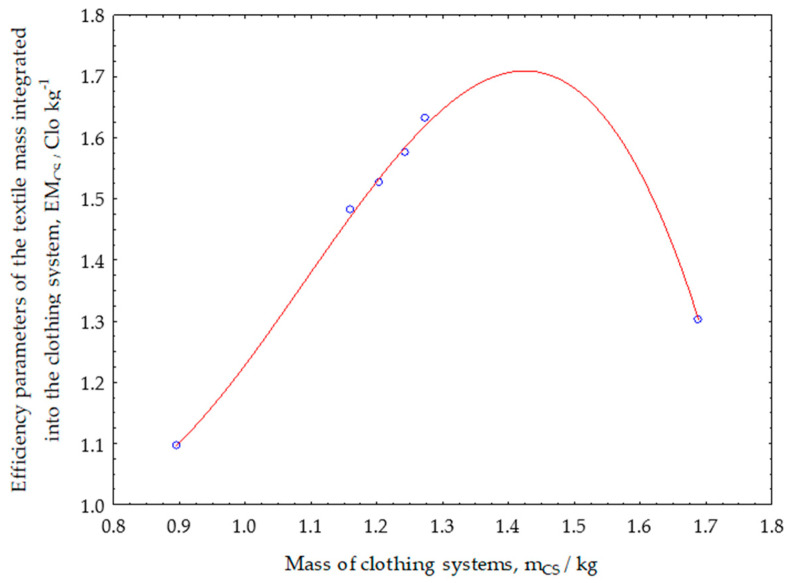
Functional dependence on changes in the values of the efficiency parameters of the textile mass integrated into the clothing systems.

The functional dependence on changes in the values of the efficiency parameters of the textile mass integrated into the clothing system is shown in Figure 8 and is determined by the expression (4). The efficiency parameter of the textile mass integrated into the thermal insulation system shows how much thermal insulation, expressed in Clo units, 1 kg of the textile thermal insulation material integrated into the clothing system can achieve. This parameter is also very important in the comparative analysis of the thermal insulation of clothing systems when it is necessary to determine the optimum efficiency of the textile mass integrated into the clothing system.

The higher the thermal insulation by using 1 kg of the integrated textile thermal insulation material, the more successful was the technical design of the thermal properties of the clothing system. Figure 8 shows that increasing the total mass of the garment system increases the efficiency of the designed system and reaches a maximum of about 1.25 kg of the total mass of the clothing system.

Thereby, 1 kg of the textile material achieves the efficiency of the integrated textile mass of 1.7 Clo kg^−1^. After maximum function dependence, any further increase in the mass of the integrated textile material leads to a decrease in the efficiency of the integrated textile mass. By using this parameter, it is also possible to determine the success of the technical design of the thermal properties of the clothing, to optimize the material consumption by quantity and price.

By the described measuring method on the thermal manikin, the evaluation of the measurement results and the introduction of three new parameters of the integration efficiency of the thermal insert, the efficiency parameter of the thermal insulation and the efficiency parameter of the textile mass integrated into the clothing system, it is possible to perform an efficient and accurate comparative analysis of the thermal insulation of multi-layer thermal inserts in the clothing designed for cold conditions. The described design method should completely replace the experience-based methods.

## 4. Conclusions

This research provides a comparative analysis of the detachable thermal insulation of multi-layer thermal inserts in the clothing system. A protective jacket for cold conditions, used by professional services, and five multi-layer thermal insulation inserts were designed for this purpose. Basic measurements were made on the thermal manikin with regard to thermal insulation, and it can be concluded that an increase in the mass of thermal insulation textile materials contributes to an increase in the thermal insulation properties of the clothing and that no further comprehensive analysis is possible. Therefore, the introduction of three new parameters for a more successful way of assessing the performance of the engineering design of thermal properties of clothing is proposed. These are the parameters of the integration efficiency of the thermal insert, the thermal insulation efficiency parameter, and the efficiency parameter of the textile mass integrated into the clothing system. Based on the above three parameters, it is possible to accurately determine the efficiency of the technical design of the thermal properties of clothing and optimal material consumption, resulting in an acceptable cost of clothing and through the ease of movement.

## 5. Patents

Dubravko Rogale, Gojko Nikolić, G.: Measuring System for Determination of Static and Dynamic Thermal Properties of Composite and Clothing. State Intellectual Property Office of the Republic of Croatia, Patent No. PK20130350 [17].

## Figures and Tables

**Table 1:** Overview of the analysed technical characteristics of the sample of the integration material.

	Outer Shell		Detachable Thermal Inserts				
	M01	M02	M03	M04	M05	M06	M07
Raw material composition	Cotton 54.5% Polyester 41.5% Metallized fibres 4%	Polyester 100%	Double-faced, diamond-shaped quilted lining: Cover fabrics: Polyester 100% Lining: Polypropylene 100% Padding: Polyester 100%				
Mass per unit area: Cover fabrics (kg m^−2^)	-	-	0.475 × 2				0.475 × 4
Mass per unit area: lining (kg m^−2^)	-	-	0.132 × 2				0.132 × 4
Mass per unit area: padding (kg m^−2^)	-	-	0.385	0.790	0.114	0.145	0.770
Mass per unit area: in total (kg m^−2^)	0.162	0.768	0.160	0.200	0.235	0.263	0.320

Remarks: M01: Inner layer of outer shell; M02: Outer layer of outer shell; M03: Material of thermal insert (TI1); M04: Material of thermal insert (TI2); M05: Material of thermal insert (TI3); M06: Material of thermal insert (TI4); M07: Material of thermal insert (TI5).

**Table 2:** Summary of designations and masses of outer shells, detachable thermal inserts, and clothing systems.

Outer Shell				Detachable Thermal Inserts			Clothing System	
Designation of the Outer Shell Material		Designation	Mass, kg	Designation of the Thermal Insert Material	Designation	Mass, kg	Designation	Mass, kg
Outer Layer	Inner Layer							
M01	M02	OS1	0.985	M03	TI1	0.176	CS1	1.161
				M04	TI2	0.220	CS2	1.205
				M05	TI3	0.259	CS3	1.244
				M06	TI4	0.289	CS4	1.274
				M07	TI5	0.704	CS5	1.689

Remarks: OS1: Outer shell of clothing systems; TI1: Thermal insert of clothing system CS1; TI2: Thermal insert of clothing system (CS2); TI3: Thermal insert of clothing system (CS3); TI4: Thermal insert of clothing system (CS4); TI5: Thermal insert of clothing system (CS5).

## References

[1] Holme’r I. (2004). Thermal manikin history and applications. Eur. J. Appl. Physiol..

[2] Jussila K. (2016). Clothing Physiological Properties of Cold Protective-Clothing and Their Effects on Human Experience.

[3] Konarska M., Sołtynski K., Sudoł-Szopińska I., Chojnacka A. (2017). Comparative Evaluation of Clothing Thermal Insulation Measured on a Thermal Manikin and on Volunteers. Fibres Text. East. Eur..

[4] Mather J.R. (1974). Climatology, Fundamentals and Applications.

[5] Eryuruk S.H. (2018). Effect of fabric layers on thermal comfort properties. AUTEX Res. J..

[6] Matusiak M. (2006). Investigation of the thermal insulation properties of multilayer textiles. Fibres Text. East. Eur..

[7] Matusia M., Sybilska W. (2016). Thermal resistance of fabrics vs. thermal insulation of clothing made of the fabrics. J. Text. Inst..

[8] Gupta D., Srivastava A., Kale S. (2013). Thermal properties of single and double layer fabric assemblies. Indian J. Fibre Text. Res..

[9] ISO (2004). Clothing-Physiological Effects-Measurement of Thermal Insulation by Means of a Thermal Manikin.

[10] Konarska M., Sołtyński K., Sudoł-Szopińska I., Młoźniak D., Chojnacka A. (2006). Aspects of Standardisation in Measuring Thermal Clothing Insulation on a Thermal Manikin. Fibres Text. East. Eur..

[11] Holand B., Nilsson H., Holmér I. (1999). Comfort temperatures for sleeping bags. Proceedings of the Third International Meeting on Thermal Manikin Testing 3IMM at the National Institute for Working Life.

[12] Rogale S.F., Benić M., Rogale D. Investigation of resistance to the passage of heat for different men’s clothing combinations. Proceedings of the 10th Scientific–Professional Symposium Textile Science & Economy.

[13] AOliveira V.M., Branco V.J., Gaspar A., Quintela D.A. Measuring Thermal Insulation of Clothing with Different Manikin Control Methods: Comparative Analysis of the Calculation Methods. Proceedings of the 7th International Thermal Manikin and Modelling Meeting.

[14] Antonnen H. Interlaboratory trial of thermal manikin. Proceedings of the 3I3M-3rd International Meeting.

[15] Bartkowiak G., Dabrowska A., Greszta A. (2020). Development of Smart Textile Materials with Shape Memory Alloys for Application in Protective Clothing. Materials.

[16] He J., Yehu L., Wang L., Ma N. (2018). On the Improvement of Thermal Protection for Temperature-Responsive Protective Clothing Incorporated with Shape Memory Alloy. Materials.

[17] Rogale D., Nikolić G. (2015). Measuring System for Determination of Static and Dynamic Thermal Properties of Composite and Clothing.

[18] Rogale D., Rogale S.F., Špelić I., Dragčević Z. (2014). Development of the Measuring System for Analysing the Thermal Properties of Clothing. Book of 7th ITC&DC 2014.

[19] Majstorović G. (2015). Determination of Thermal Properties of Special Purpose and Intelligent Clothing During Their Technical Design. Ph.D. Thesis.

